# Comparative Analysis of the Hemostasiological Profile in Sheep and Patients with Cardiovascular Pathology as the Basis for Predicting Thrombotic Risks During Preclinical Tests of Vascular Prostheses

**DOI:** 10.17691/stm2021.13.1.06

**Published:** 2021-02-28

**Authors:** O.V. Gruzdeva, E.E. Bychkova, T.Yu. Penskaya, A.A. Kuzmina, L.V. Antonova, L.S. Barbarash

**Affiliations:** Head of the Laboratory of Homeostasis Research, Research Institute for Complex Issues of Cardiovascular Diseases, 6 Sosnovy Blvd, Kemerovo, 650002, Russia; Research Assistant, Laboratory of Homeostasis Research, Research Institute for Complex Issues of Cardiovascular Diseases, 6 Sosnovy Blvd, Kemerovo, 650002, Russia; Junior Researcher, Laboratory of Homeostasis Research, Research Institute for Complex Issues of Cardiovascular Diseases, 6 Sosnovy Blvd, Kemerovo, 650002, Russia; Junior Researcher, Laboratory of Homeostasis Research, Research Institute for Complex Issues of Cardiovascular Diseases, 6 Sosnovy Blvd, Kemerovo, 650002, Russia; Head of the Laboratory of Cell Technologies, Research Institute for Complex Issues of Cardiovascular Diseases, 6 Sosnovy Blvd, Kemerovo, 650002, Russia; Professor, Academician of the Russian Academy of Sciences, Chief Researcher, Research Institute for Complex Issues of Cardiovascular Diseases, 6 Sosnovy Blvd, Kemerovo, 650002, Russia

**Keywords:** hemostasiological profile, prediction of thrombotic risks, antithrombotic drugs

## Abstract

**The aim of the investigation** was to study the details of hemostasiological profile in sheep and patients with coronary heart disease (CHD) and to find the possibility of predicting thrombotic risks during preclinical tests of vascular prostheses on a large laboratory animal model.

**Materials and Methods.:**

The functional activity of platelets was measured in platelet-rich plasma with inductors: ADP, epinephrine, collagen. Prothrombin activity, international normalized ratio, activated partial thromboplastin time (APTT), thrombin time, fibrinogen concentration, antithrombin III and protein C activity, fibrinolysis were determined in blood plasma. Changes in clot formation and viscoelastic properties of clots were assessed using thromboelastography.

**Results.:**

Significant differences were found in the hemostasiological profile of sheep and CHD patients. Sheep platelets had increased response to ADP induction and practically no response to epinephrine induction; collagen-induced aggregation was comparable in the study groups. Coagulation hemostasis of sheep was characterized by increased activity of the prothrombin complex, shortened thrombin time, while APTT and fibrinogen values remained comparable. At the same time, sheep exhibited a significant decrease in the activity of anticoagulant and fibrinolytic systems as compared to CHD patients. When assessing dynamic changes in clot formation, it was observed that initiation phase was faster in animals, while clot density exceeded that in patients.

**Conclusion.:**

The hemostasiological profile of sheep is characterized by the increased speed of thrombus formation, greater strength of the formed clot, and lower lysis ability as compared to CHD patients. The revealed details of the hemostasiological profile of sheep can be potential targets for therapy with antithrombotic drugs that minimize thrombotic risks in preclinical testing of vascular prostheses.

## Introduction

Thrombosis of vascular grafts, caused by aggressive intervention in the hemostatic system, affects the outcomes of cardiac surgery aimed at restoring the blood flow and improving the quality of life in patients with coronary heart disease (CHD) [1, 2]. A similar problem often occurs when carrying out preclinical tests of vascular prostheses in animal models [3]. Notably, some of them (for example, sheep model) are the most aggressive in terms of identifying hemostasiological risks associated with failure of tested vascular prostheses. In most cases, using this model makes it possible to achieve implanted prostheses patency of a little more than 60% [4, 5]. To date, the causes of aggressive response of hemostatic system of sheep during preclinical tests of vascular prostheses are still understudied.

Comparative studies in humans, sheep, pigs, rabbits, rats, and dogs using standard coagulation tests as well as clotting factor analysis and thromboelastography have shown that the human blood coagulation system is greatly similar to the sheep blood coagulation system [6]. Sheep blood has an increased tendency to coagulation, but the nature of this phenomenon is not fully understood. Besides, data defining the “normal” parameters of sheep blood for both classical and new methods of assessing coagulation are currently insufficient. To date, studies are limited by the small sample size and identification of only a narrow range of coagulation parameters [7]. Finding the reasons for activation of hemostatic potential before surgery and in response to implantation of vascular prostheses being developed as well as possible contribution of individual aspects of the recipient’s hemostasis system to implantation outcomes requires in-depth investigation that will subsequently help to extrapolate the obtained results to humans.

**The aim of the investigation** was to study the details of hemostasiological profile in sheep and patients with coronary heart disease and to find the possibility of predicting thrombotic risks on a large laboratory animal model during preclinical tests of vascular prostheses.

## Materials and Methods

The study was carried out at the Research Institute for Complex Issues of Cardiovascular Diseases (Kemerovo, Russia).

The subject of the research was whole blood of animals (sheep, n=50) and that of patients with the established diagnosis of CHD (n=86), receiving double antiplatelet therapy (acetylsalicylic acid and clopidogrel). The study protocol was approved by the local Ethics Committee and compliant with the principles of the Declaration of Helsinki (2013). Informed consent was obtained from every patient.

Care and use of experimental animals complied with the international guidelines set forth in the “Guide for the Care and Use of Laboratory Animals” (National Research Council, 2011), and the ethical principles established by the European Convention for the Protection of Vertebrate Animals Used for Experimental and Other Scientific Purposes (Strasburg, 2006).

Quick’s prothrombin time and the international normalized ratio (INR) were determined in venous blood plasma of animals and CHD patients using the ACL 7000-1 coagulometer (Instrumentation Laboratory, USA). Activated partial thromboplastin time (APTT), Clauss fibrinogen, thrombin time, protein C, antithrombin III were determined using Ceveron alpha analyzer (Technoclone GmbH, Austria) in accordance with the manufacturer’s instructions. Fibrinolytic activity was measured in plasma using the XIIa-kallikrein-dependent fibrinolysis test also in accordance with the manufacturer’s instructions (Technology-Standard, Russia).

Thromboelastography was performed on the TEG 5000 Thrombelastograph analyzer (Haemonetics, USA). The following parameters were analyzed: clotting time (*R* (min)), clot formation time (*K* (min)), maximum amplitude (MA (mm)), and angle (°). The *R* interval is the time elapsed from the start of the test to the formation of the first fibrin strands. This interval clearly coincides with the first phase of blood coagulation (initiation). *R* and *K* characterize activation and dynamics of clot formation. Angle shows growth rate of the fibrin network and its structuring (increase in clot strength). MA provides clot strength information.

The platelet aggregation activity was determined on the Helena AggRAM aggregometer (Helena BioSciences Europe, UK), measuring the degree (max (%)) and time (max time (s)) of maximum platelet aggregation with inducers: ADP (1.25 and 2.5 μg/ml), epinephrine (5 μg/ml), collagen (100 μg/ml).

**Statistical processing of the results** was performed using Statistica 10.0 software package. The Kolmogorov–Smirnov test was used to assess population distribution pattern based on sample data. The results were presented as median (Me) and the 25^th^ and 75^th^ percentiles (Me [Q1; Q3]). Comparison of independent groups was performed using the Mann–Whitney U test. Differences were considered statistically significant at p<0.05.

## Results

Aggregatogram results showed that ADP-induced platelet aggregation recorded in sheep exceeded the indicators of CHD patients. For example, the degree and time of maximum aggregation in animals were 1.5 and 1.8 times higher than in CHD patients at ADP concentration of 1.25 μg/ml and 1.3 and 1.6 times, respectively, at ADP concentration of 2.5 μg/ml (Table 1). Animal platelets, unlike platelets of patients, had practically no response to epinephrine induction. In both study groups, there were no statistically significant differences in the parameters of collagen-induced platelet aggregation (see Table 1). The number of platelets in animal blood plasma was 2 times higher than in the blood plasma of CHD patients — 470 (462; 485)**·**1012/L and 229 (218; 286)**·**1012/L, respectively.

**Table 1:** Aggregatogram parameters in sheep and CHD patients (Me [Q1; Q3])

Groups	ADP 1.25 μg/ml		ADP 2.5 μg/ml		Epinephrine		Collagen	
	max (%)	max time (s)	max (%)	max time (s)	max (%)	max time (s)	max (%)	max time (s)
Sheep	92.6	432.5	93.6	484.5	3.6	467.0	85.1	313.0
	[87.4; 120.3]	[322.5; 523.0]	[91.2; 113.8]	[401.5; 522.0]	[0.9; 4.8]	[137.0; 585.0]	[41.2; 97.3]	[186.0; 522.0]
CHD patients	60.1	244.0	72.2	298.5	84.4	530.0	86.6	398.0
	[44.8; 67.1]^*^	[126.0; 288.5]^*^	[66.8; 76.2]^*^	[255.5; 353.0]^*^	[81.5; 95.3]^*^	[426.0; 547.0]	[73.7; 88.7]	[336.0; 481.0]

^*^ Statistically significant differences between the groups, p<0.05.

When analyzing the coagulogram data, it was found that plasma prothrombin activity was 1.2 times higher in animals than in CHD patients. Calculation of INR showed that this indicator was 1.2 times lower in sheep as compared to humans (Table 2).

**Table 2:** Coagulogram indices in sheep and CHD patients (Me [Q1; Q3])

Groups	Prothrombin (%)	INR	APTT (s)	Thrombin time (s)	Fibrinogen (g/L)
Sheep	104.0 [82.7; 114.0]	0.98 [0.89; 1.22]	26.2 [23.9; 30.5]	12.3 [11.7; 13.7]	2.2 [1.7; 3.3]
CHD patients	89.0 [80.0; 93.4]^*^	0.92 [0.89; 0.94]	29.1 [28.1; 30.1]	14.8 [14.2; 16.0]^*^	3.0 [2.8; 3.6]

^*^ Statistically significant differences between the groups, p<0.05.

APTT values in the study groups were comparable. Thrombin time was found to be 1.2 times shorter in the blood plasma of animals as compared to the data obtained in patients (p<0.05). Analysis of fibrinogen concentration in the blood plasma of both study groups showed no statistically significant differences (see Table 2). Fibrinolytic activity of sheep blood plasma was 60% lower than in CHD patients (p<0.05).

Activity of natural anticoagulants — antithrombin III and protein C — was 1.2 and 3.5 times lower in the blood plasma of animals than in CHD patients (p<0.05) (see the Figure).

**Figure:**
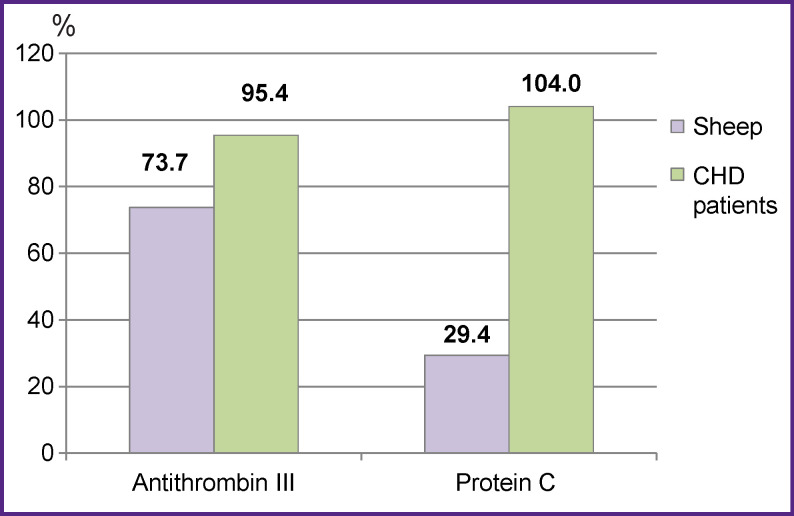
Plasma antithrombin III and protein C activity in sheep and CHD patients The differences between the groups are statistically significant, p<0.05

Thromboelastogram was performed to assess thrombus formation kinetics and fibrin clot density. The *R* interval in whole blood of animals was 1.4 times less compared to the same parameter in patients (p<0.05). The *K* value had no significant differences in the study groups. In sheep, the angle values characterizing functional activity of fibrinogen in the blood exceeded those of CHD patients by 1.1 times (p<0.05). The MA value in sheep was 1.1 times higher than in patients (Table 3).

**Table 3:** Thromboelastogram indices in sheep and CHD patients (Me [Q1; Q3])

Groups	*R* (min)	*K* (min)	Angle (°)	MA (mm)
Sheep	4.4	1.1	75.1	77.1
	[4.0; 4.7]	[0.1; 1.1]	[73.9; 76.9]	[75.4; 80.5]
CHD patients	6.2	1.2	71.4	70.1
	[6.0; 6.4]^*^	[1.1; 1.2]	[70.8; 72.8]^*^	[70.2; 73.9]^*^

^*^ Statistically significant differences between the groups, p<0.05.

## Discussion

Studies in animal models have been carried out in all areas of medicine, but the ability to extrapolate these data to patients is limited. This article provides a comparative analysis of parameters reflecting the hemostatic system of sheep and patients with cardiovascular diseases. This category of patients was selected for the study because they are the potential consumers of vascular grafts actively tested in sheep. It was found in our study that the parameters of the hemostatic system of sheep and CHD patients were largely comparable, though significant differences were observed in some individual components. In the work, we used techniques that allowed us to determine both local and integral changes in the hemostatic system. Local tests make it possible to pinpoint the functional activity of platelets, the state of the internal or external pathway in laboratory conditions (*in vitro*). The use of integral techniques better reflects blood coagulation process *in vivo* [8].

When studying platelet hemostasis, we revealed differences in the functional activity of platelets in the study groups. ADP-induced platelet aggregation values recorded in sheep were higher than in CHD patients. The obtained study results can be attributed to the increased content of platelets in the blood plasma of animals, which is accompanied by increased activation of purinergic receptors. As a result, the rate of ADP binding to P2X_1_ receptor increases mediating more rapid extracellular calcium influx, which leads to alteration in platelet shape and aggregation of platelets with each other via P2Y receptor [9].

In our study, sheep platelets practically did not respond to epinephrine induction. The mechanism underlying realization of epinephrine effect and development of aggregation is stimulation of α2-adrenergic receptors associated with Gz-protein, which leads to formation of thromboxane A2 from arachidonic acid [10, 11]. Since epinephrine is a weak inducer, an increased platelet count is likely to result in increased proportion of non-aggregated platelets and very low aggregatometry values, which has been demonstrated.

Collagen-induced platelet aggregation had no statistically significant differences in the groups under study. As long as several platelet receptors are involved in realization of collagen effects, it can be suggested that one of the pathways promoting aggregation is less prominent in sheep. Supposedly, expression of integrin α2β1 and/ or glycoprotein VI (GPVI) receptors permitting collagen to bind directly to the platelet surface is reduced [9].

Several significant differences in the plasma-coagulation hemostasis of the study groups were revealed by performing local tests. According to the results obtained, prothrombin activity in the blood plasma of animals was higher, while the INR values were lower as compared to those of CHD patients. Local tests are applicable to assess the external blood coagulation pathway. The external pathway is a shorter pathway of coagulation hemostasis, which is influenced mainly by factor VII level in the blood plasma. There are studies reporting prolonged prothrombin time in sheep due to low levels of factor VII. There is some evidence that the level of factor VII is only 13% of the normal values typical for human plasma, other sources report it to be 36 to 45.5% [7]. In our study, CHD patients took antithrombotic drugs, so their plasma prothrombin activity was lower, even with allowance for the lower factor VII level in sheep. Despite the statistically significant differences between the study groups, both parameters did not go beyond the established reference intervals: 70–130% for prothrombin activity, 0.85–1.25 for INR.

Comparable APTT values in blood plasma of sheep and CHD patients correspond to the reference interval (26–36 s), which confirms the absence of significant differences in the internal pathway of the hemostatic system between the study groups [6].

In the blood plasma of sheep, thrombin time shortening is observed at comparable values of fibrinogen. As long as thrombin time is a laboratory parameter characterizing the rate of converting fibrinogen to fibrin when thrombin is added to the plasma, our data suggest the increased prothrombin activity in the blood plasma of sheep. In this case, the thrombin time in the blood plasma of CHD patients does not fall within the reference interval of 12.0–14.0 s.

Multiple decrease in the activity of physiological anticoagulants in the blood plasma of sheep is of particular interest. We found antithrombin III to be reduced by 13% and protein C — by 72%. Antithrombin III is known to act as an anticoagulant forming stable complexes with thrombin, factor Xa, and other serine proteases of the internal pathway. Complex formation involves interaction between the reactive site of antithrombin III and the active sites of its targets. Antithrombin III is a heparin cofactor. Heparin binds to antithrombin III through a pentasaccharide sequence, inducing conformational changes that promote antithrombin-mediated inhibition of these coagulation factors [12]. Most likely, the decrease in antithrombin III activity in sheep is due to interspecies differences in the substance structure as compared to humans. Human antithrombin and that of sheep are known to be 89% similar in the amino acid sequence. Unlike human antithrombin III consisting of a single polypeptide chain with 432 amino acids, sheep antithrombin III has additional amino acid at position 6.

Protein C system regulates the activity of factor VIIIa (FVIIIa) and factor Va (FVa), cofactors activating factor X and prothrombin. This system consists of membrane-bound and circulating proteins that assemble into multi-molecular complexes on the cell surface. Vitamin K-dependent protein C, the key component of the system, circulates in the blood as serine protease proenzyme. It is activated on the surface of endothelial cells by thrombin associated with membrane protein thrombomodulin. Endothelial protein C receptor (EPCR) further stimulates activation of protein C. Activated protein C, together with its cofactor protein S, inhibits coagulation by inactivating FVIIIa and FVa on the surface of negatively charged phospholipid membranes [13].

The reasons for the reduced protein C activity in the blood plasma of sheep are yet to be studied. This multiple decrease in the activity of natural anticoagulants in the blood plasma of animals is likely to cause hypercoagulation.

Analysis of the fibrinolytic activity of blood plasma makes it possible to assess the state of the internal and external mechanisms of plasminogen activation, plasmin formation, and fibrin clot lysis. Fibrinolysis efficiency is largely influenced by clot structure, fibrinogen polymorphism, the rate of thrombin generation, platelet reactivity, and the overall biochemical environment [14]. According to our data, sheep have significantly lower plasma fibrinolytic activity as compared to CHD patients, which may be due to the differences in the above factors. Low fibrinolytic activity of sheep plasma may be another cause of increased thrombus formation in animals.

However, as long as all of the above tests are performed in plasma, they are endpoint analyses that cannot assess dynamic changes in the viscoelastic properties of a blood sample. In earlier experiments, rotational thromboelastometry was used to eliminate these shortcomings [6, 15]. In our study, we used a thromboelastogram to assess not only the plasmic hemostasis component, but also the cellular component.

Thromboelastograms exhibited shorter *R* interval in animals as compared to the same indicator in CHD patients; therefore, thrombus formation process was faster in the blood of animals. The angle values reflecting the increase in clot strength and characterizing functional fibrinogen activity in whole blood were higher in sheep than in CHD patients, while there were no differences between the groups in plasma fibrinogen level. MA was higher in sheep. The MA indicator characterizes the state of fibrinogen and platelets that have unequal influence on thrombus properties with 80% of it exerted by platelets. The increase in clot density might be attributed to the significantly higher platelet count in sheep.

Thus, we found significant differences in the hemostasiological profile of sheep and CHD patients: sheep platelets were characterized by increased response to ADP induction, but they practically did not respond to epinephrine induction. Plasma-coagulation hemostasis of sheep was characterized by increased activity of the prothrombin complex, shortened thrombin time, while APTT and fibrinogen values remained comparable in the study groups. At the same time, sheep exhibited a significant decrease in the activity of anticoagulant and fibrinolytic systems as compared to CHD patients. Analysis of dynamic changes in clot formation showed that initiation phase was faster in animals, while clot density exceeded that in CHD patients.

## Conclusion

The hemostasiological profile of sheep is characterized by the increased speed of thrombus formation, greater strength of the formed clot, and lower lysis ability as compared to CHD patients. The revealed details of the hemostasiological profile of sheep can be potential targets for therapy with antithrombotic drugs that minimize thrombotic risks in preclinical testing of vascular prostheses on animal models.
